# Efficient One‐Step Production of 7*S*,17*S*‐ and 10*S*,17*S*‐Dihydroxydocosahexaenoic Acids by a Double‐Oxygenating 15*S*‐Lipoxygenase From *Chlamydomonas incerta*

**DOI:** 10.1002/bit.28997

**Published:** 2025-04-15

**Authors:** Hyun‐Ah Park, Jin Lee, Deok‐Kun Oh

**Affiliations:** ^1^ Department of Bioscience and Biotechnology Konkuk University Seoul Republic of Korea

**Keywords:** arachidonate 15*S*‐lipoxygenase, *Chlamydomonas incerta*, dihydroxy fatty acids, protectin DX, resolvin D5

## Abstract

Specialized pro‐resolving mediators (SPMs), such as resolvin D5 (7*S*,17*S*‐dihydroxydocosahexaenoic acid, 7*S*,17*S*‐DiHDHA; RvD5) and protectin DX (10*S*,17*S*‐DiHDHA; PDX), are critical in resolving inflammation in humans. In this study, a unique double‐oxygenating 15*S*‐lipoxygenase (15*S*‐LOX) from the alga *Chlamydomonas incerta* was identified and characterized for its ability to simultaneously produce RvD5 and PDX from docosahexaenoic acid (DHA). Recombinant *Escherichia coli* expressing the *C. incerta* 15*S*‐LOX demonstrated enhanced RvD5 and PDX production under the following optimized reaction conditions: pH 8.0, 25°C, 0.5 g dry cells/L, 7.0 mM DHA, 2.0% (w/v) PVP, 2.0% (v/v) DMSO, and 200 mM cysteine used as a reductant. This one‐step biocatalytic process produced 2.91 mM (1.05 g/L) RvD5 and 2.18 mM (0.78 g/L) PDX in 90 min, with a total of 5.09 mM (1.83 g/L) and a total conversion yield of 79.6% (w/w). Compared to previously reported two‐step biocatalytic processes, this one‐step process significantly enhanced the production of particular PDX with improved productivity and simplicity. Structural analysis identified residues Phe667, Ile705, and Leu713 as regioselectivity modulators for the second oxygenation step. This study demonstrates the efficiency and industrial potential of the double‐oxygenating LOX as a biocatalyst for simultaneously producing RvD5 and PDX.

## Graphical abstract

Biotransformation pathway from polyunsaturated fatty acids into dihydroxy fatty acids as lipid mediators by *Escherichia coli* expressing *Chlamydomonas incerta* 15*S*‐lipoxygenase with double oxygenating activity. The 15*S*‐lipoxygenase was the most efficient for the simultaneous production of resolvin D5 (7*S*,17*S*‐dihydroxydocosahexaenoic acid, 7*S*,17*S*‐DiHDHA; RvD5) and protectin DX (10*S*,17*S*‐DiHDHA; PDX) as specialized pro‐resolving mediators (SPMs) from docosahexaenoic acid (DHA).

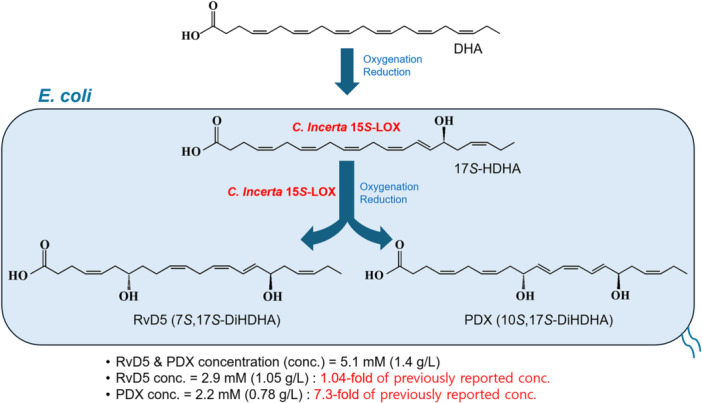

## 1 Introduction

Single‐ and double‐oxygenating lipoxygenase (LOXs) catalyze the one‐step and two‐step oxygenation of C20‐ and C22‐polyunsaturated fatty acids (PUFAs) containing 1,4‐*Z*,*Z*‐pentadiene structures, including arachidonic acid (ARA), eicosapentaenoic acid (EPA), n‐3 docosapentaenoic acid (n‐3 DPA), and docosahexaenoic acid (DHA), to produce their C20‐ and C22‐monohydroxy fatty acids (MonoHFAs) and dihydroxy fatty acids (DiHFAs) with *Z*,*E*‐conjugated dienes, respectively, in the presence of a reductant or under natural conditions (An et al. 2021).

DiHFAs derived from EPA, n‐3 DPA, and DHA are classified as specialized pro‐resolving mediators (SPMs). SPMs are generated by M2 macrophages in humans and are crucial in resolving inflammation and infection in humans (Chávez‐Castillo et al. 2021; Yamada et al. 2021). Among SPMs, resolvin D5 (RvD5; 7*S*,17*S*‐dihydroxydocosahexaenoic acid, 7*S*,17*S*‐DiHDHA) improves phagocytosis, enhances bacterial killing, reduces UVB‐induced skin damage, and alleviates type 1 diabetes (Chiang et al. 2012; Chun et al. 2020; Leão et al. 2022; Saito et al. 2024), while protectin DX (PDX; 10*S*,17*S*‐DiHDHA) inhibits reactive oxygen species production, suppresses fibroblast proliferation, and induces an antioxidant effect (Dai et al. 2015; Lagarde et al. 2020; Yang et al. 2020).

SPMs are classified into four main families: lipoxins, protectins, maresins, and resolvins (An et al. 2021). Lipoxins are TriHFAs with 5‐ and 15‐hydroxyl groups derived from ARA; protectins are DiHFAs with 10‐ and/or 17‐hydroxyl groups derived from DHA and n‐3 DPA; maresins are DiHFAs with 7‐ and/or 14‐hydroxyl groups, also derived from DHA and n‐3 DPA; and resolvins are DiHFAs and TriHFAs with 5‐, 15‐, and/or 18‐hydroxyl groups derived from EPA or with 7‐ and/or 17‐hydroxyl groups derived from DHA and n‐3 DPA.

SPMs have primarily been synthesized using chemical methods. However, these methods often involve heavy metals, such as Pd (PPh_3_)_2_Cl_2_ and CrCl_2_, and require complex multi‐step reaction sequences (20−30 steps), resulting in environment‐unfriendly, costly, and inefficient processes (Aursnes et al. 2014; Nshimiyimana et al. 2023; Ogawa et al. 2017; Rodríguez and Spur 2005). To address these limitations, two types of distinct regioselective 66 single‐oxygenating LOXs and one double‐oxygenating LOX have been used in quantitatively producing SPMs. The concentrations and productivities of SPMs obtained via one‐step biocatalytic processes using double‐oxygenating LOXs (T. H. Kim et al. 2021; J. Lee, Ko, Park, et al. 2024; J. Lee et al. 2023; Oh et al. 2022) have been higher than those achieved via sequential biocatalytic processes with two distinct regioselective single‐oxygenating LOXs (T. E. Lee, Ko, Shin, et al. 2024; Shin et al. 2022), highlighting the superior efficiency of double‐oxygenating LOXs for SPM production. However, the use of double‐oxygenating LOXs to the quantitatively biotechnological production of PDX has not yet been reported.

**Figure 1:**
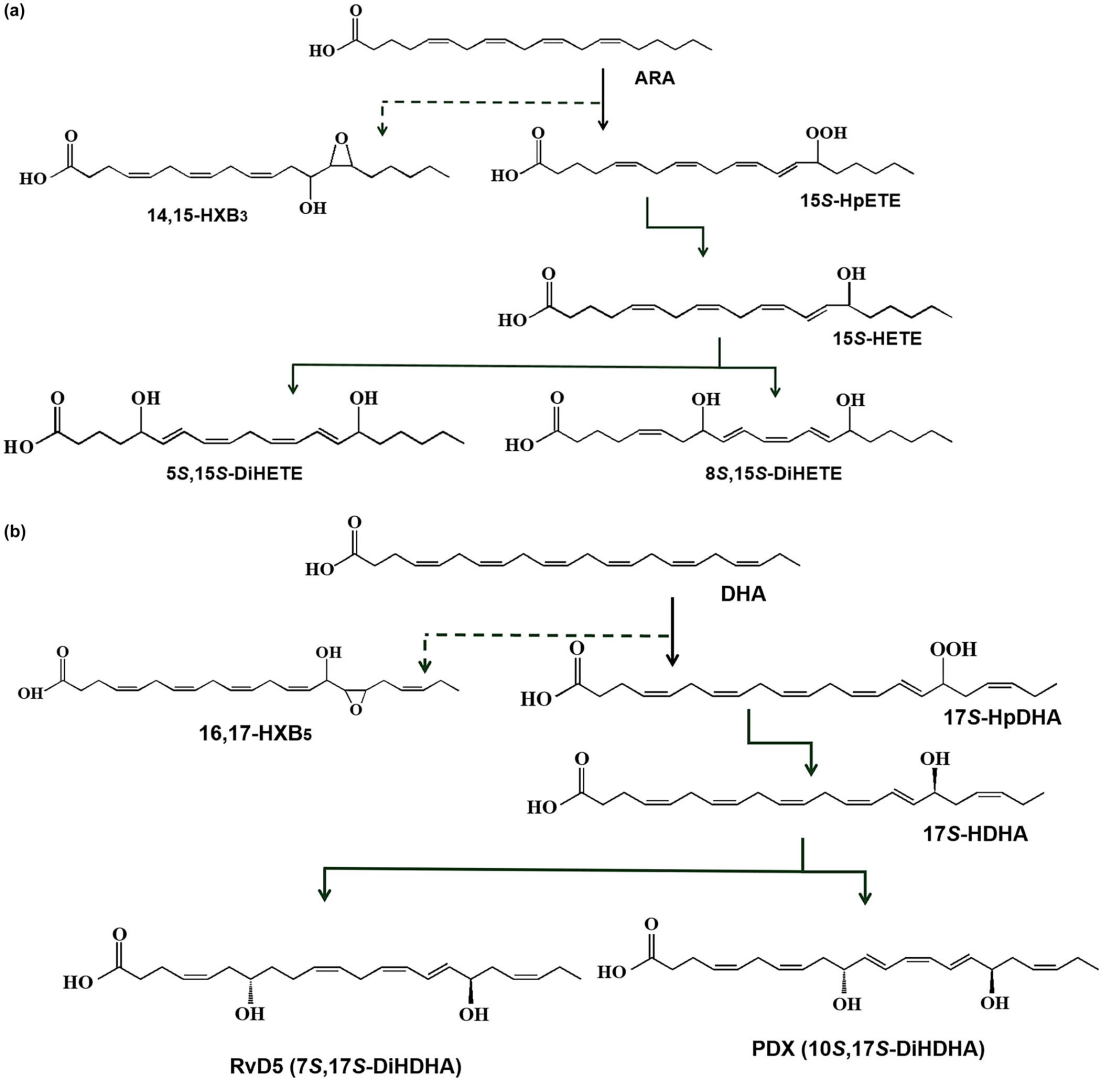
Biosythetic pathways of ARA and DHA to 5*S*,15*S*‐ and 8*S*,15*S*‐DiHETEs and RvD5 and PDX by *Escherichia coli* expressing 15*S*‐LOX from *Chlamydomonas incerta*, respectively. (a) Biosythetic pathway from ARA to 5*S*,15*S*‐ and 8*S*,15*S*‐DiHETEs via 14,15‐HXB_3_ and 15*S*‐HpETE. (b) A biosynthetic pathway from DHA to RvD5 and PDX via 16,17‐HXB5 and 17*S*‐HpDHA. 14,15‐HXB_3_, 14,15‐hepoxilin B_3_; 13‐hydroxy‐14,15‐epoxy‐eicosatrienoic acid; 16,17‐HXB_5_, 16,17‐hepoxilin B_5_; 15‐Hydroxy‐16,17‐epoxy‐4,7,10,13,19‐docosapentaenoic acid.

This study focused on identifying and characterizing a putative LOX from *Chlamydomonas incerta* as a double‐oxygenating ARA 15*S*‐LOX capable of the quantitative production of 5*S*,15*S*‐DiHETE (RvD5) and 8*S*,15*S*‐DiHETEs (PDX) from ARA and DHA in a single step (Figure 1). The reaction conditions, including pH, temperature, cell, substrate, polymer, and solvent concentrations, were optimized using *Escherichia coli* expressing *C. incerta* 15*S*‐LOX to enhance SPM production. These optimizations improved RvD5 and PDX yields, with PDX production showing significantly enhancement compared to previously reported two‐step processes. Homology modeling and structural analysis also identified key residues involved in regioselectivity for the second oxygenation step catalyzed by *C. incerta* 15*S*‐LOX.

## 2 Materials and Methods

### 2.1 Materials

ARA, DHA, and EPA standards were purchased from Sigma‐Aldrich (St. Louis, MO, USA). Standards for 5*R*‐, 8*R*‐, 15*S*‐, and 15*R*‐hydroxyeicosatetraenoic acid (HETE) and 5*S*,15*S*‐ and 8*S*,15*S*‐dihydroxyeicosatetraenoic acid (DiHETE) were obtained from Cayman Chemical (Ann Arbor, MI, USA). Standards for 5*R*,15*S*‐, 8*R*,15*S*‐, 5*R*,15*R*‐, and 8*R*,15*R*‐DiHETE were prepared by purifying the reaction products obtained from the conversion of reagent‐grade 5*R*‐ or 8*R*‐HETE using 15*S*‐LOX from *Archangium violaceum* and 15*R*‐LOX from *Sorangium cellulosum*. These reactions were performed at 25°C in 50 mM HEPPS buffer (pH 8.5), containing 0.3 mM of 5*R*‐ or 8*R*‐HETE, 0.2 mg/mL enzyme, and 200 mM cysteine as a reducing agent for 120 min. The reaction mixtures were purified into DiHETE standards using preparative high‐performance liquid chromatography (Prep‐HPLC) with absorbent resin (SP825), as previously described (J. Lee, Ko, Park, et al. 2024).

### 2.2 Gene Synthesis and Cloning

Thirteen putative LOX genes from green algae, including the gene encoding *C. incerta* LOX (UniProt number, A0A835WEM6), were synthesized by L&C Bio (Seoul, Republic of Korea). The synthesized genes were cloned into the expression vector pET‐28a (+) and subsequently transformed into *E. coli* ER2566.

### 2.3 Cell Culture Conditions

*E. coli* cells were cultured in a 2 L baffled flask containing 450 mL of Luria–Bertani medium supplemented with 0.1 mM kanamycin to express the *C. incerta* 15*S‐*LOX. Cultures were incubated at 37°C under agitation at 200 rpm until the optical density at 600 nm (OD_600_) reached approximately 0.7. Protein expression was induced by adding 0.1 mM isopropyl‐β‐d‐thiogalactopyranoside. The culture was then incubated at 16°C under agitation at 150 rpm for an additional 16 h to maximize the expression of *C. incerta* 15*S‐*LOX.

### 2.4 Enzyme Purification

*E. coli* cells expressing *C. incerta 15S‐LOX* were harvested by centrifugation at 13,000 × *g* for 20 min at 4°C. The cell pellet was washed with saline and disrupted by 20 min of sonication. Cellular debris was removed by a second centrifugation at 13,000 × *g* for 10 min at 4°C. The supernatant was loaded onto a His‐Trap HP affinity chromatography column pre‐equilibrated with 50 mM phosphate buffer (pH 8.0) containing 300 mM NaCl, using a fast protein liquid chromatography system (Bio‐Rad, Hercules, CA, USA). The bound protein was eluted at 1 mL/min with 50 mM Tris‐HCl buffer (pH 8.0) containing 250 mM imidazole. The fraction exhibiting LOX activity was collected and used as the purified enzyme.

### 2.5 Molecular Weight Determination

The subunit molecular weight of the purified 15*S*‐LOX from *C. incerta* was determined by SDS‐PAGE using a protein size marker, and the protein bands were visualized by Coomassie blue staining. The enzyme solution and reference proteins, including ovalbumin (44 kDa), conalbumin (75 kDa), aldolase (158 kDa), and ferritin (440 kDa), were applied to gel filtration chromatography using a Sephacryl S‐300 HR column to determine the total molecular weight of the native *C. incerta* 15*S*‐LOX, which was calculated by comparing its elution time with that of the reference proteins.

### 2.6 Determination of Specific Activity and Kinetic Parameters

The specific activity and kinetic parameters of *C. incerta* 15*S*‐LOX for ARA, EPA, and DHA were determined using a UV spectrophotometer. The product formation was monitored by measuring the increase of absorbance at 234 nm, which corresponds to hydroperoxide formation. The reactions were performed in 50 mM HEPPS buffer (pH 7.5) at 35°C for 5 min by varying the substrate concentration from 0.05 to 0.2 mM and the enzyme concentration from 0.2 to 0.5 mg/mL. The kinetic parameters were calculated by fitting the data to the Michaelis–Menten equation using nonlinear regression analysis in GraphPad Prism 8.0 (Sand Diego, CA, USA).

### 2.7 Effects of pH and Temperature

The effects of pH and temperature on the production of DiHFAs by purified *C. incerta* 15*S*‐LOX and *E. coli* cells expressing *C. incerta* 15*S*‐LOX were investigated. The recombinant *E. coli* cells were cultured, harvested, and frozen at −80°C to permeabilize the cell membrane. These frozen cells were thawed and then used for the production of DiHFAs. The pH‐dependent activity was assessed by varying the pH from 7.0 to 9.0, using 50 mM HEPES buffer (pH 7.0−7.5), HEPPS buffer (pH 7.5−8.5), and CHES buffer (pH 8.5−9.0), at 35°C for 30 min using the enzyme and 25°C for 60 min using the cells. The temperature‐dependent activity was evaluated by varying the temperature from 15°C to 40°C at pH 7.5 and 8.0. The reactions were performed with 0.5 mg/mL purified enzyme or 0.5 g/L dry cells using 1.0 mM ARA or DHA as a substrate and 200 mM cysteine under agitation at 200 rpm for 30 min.

### 2.8 Optimization of PVP and DMSO Concentrations

The optimization of PVP and DMSO concentrations for RvD5 and PDX production were investigated using *E. coli* cells expressing *C. incerta* 15*S‐*LOX. The reactions were performed at 25°C in 50 mM HEPPS (pH 8.0) buffer containing 1.0 mM DHA, 0.5 g/L dry cells, 2.0% (v/v) DMSO with PVP or 2.0% (w/v) PVP with DMSO, and 200 mM cysteine for 60 min by varying the PVP concentration from 1.0% to 10% (w/v) or the DMSO concentration from 1.0% to 10% (v/v), respectively.

### 2.9 Optimization of Cell and Substrate Concentrations

The optimal concentrations of recombinant cells and substrate for DiHDHA production were determined. Reactions were performed at 25°C in 50 mM HEPPS (pH 8.0) buffer containing 7.0 mM DHA with dry cells or 0.5 g/L dry cells with DHA, 2.0% (w/v) PVP, 2.0% (v/v) DMSO, and 200 mM cysteine for 60 min. Cell concentration was varied from 0.1 to 1.0 g/L dry cells, and DHA concentration was adjusted from 0.5 to 8.0 mM to identify the optimal conditions.

### 2.10 Production of DiHDHAs Under Optimized Conditions

The time‐course bioconversion for DiHDHA production using *E. coli* expressing *C. incerta* 15*S‐*LOX was performed at 25°C in 50 mM HEPPS buffer (pH 8.0) containing 7.0 mM DHA, 0.5 g/L dry cells, 2.0% (w/v) PVP, 2.0% (v/v) DMSO, and 200 mM cysteine. The reaction was performed in a 100 mL baffled flask with a 10 mL reaction volume and shaken at 200 rpm for 90 min.

### 2.11 Determination of Specific Rotation

The specific rotations of the *S. cellulosum* 15*R*‐LOX DiHETE products, 5*S*,15*S*‐, 8*S*,15*S*‐, 5*R*,15*R*‐, and 8*R*,15*R*‐DiHETEs, were measured at 20°C using a polarimeter at 589 nm in 0.10−0.19 mg/mL.

### 2.12 HPLC Analysis

PUFAs, MonoHFAs, and DiHFAs were analyzed using an HPLC system with a Nucleosil C18 column (Phenomenex, Torrance, CA, USA). The reverse‐phase column was maintained at 30°C and eluted with a solvent gradient from solvent 1 (acetonitrile/water/acetic acid, 50:50:0.1, v/v/v) to solvent 2 (acetonitrile/water/acetic acid, 100:0:0.1, v/v/v) (J. Lee et al. 2020). The chirality of the MonoHFAs and DiHFAs was determined at 235 and 270 nm, respectively, using a Lux Amylose‐1 column (Phenomenex).

### 2.13 LC‐MS/MS Analysis

MonoHFAs and DiHFAs were analyzed using an LCQ Deca XP Plus ion trap mass spectrometer (Thermo Fisher Scientific, Pittsburgh, PA, USA) at the NICEM facility (Seoul National University, Seoul, Republic of Korea). Electrospray ionization was performed under the following conditions: 275°C, voltage of 5 kV, pressure of 30 psi, and capillary voltage of 20 V in the negative ion mode. The average scan time was set to 0.01 with 0.02 min allocated for polarity switching. The precursor ions were fragmented at a collision energy of 35%, maintaining 35% abundance for optimal ion detection.

### 2.14 Homology Modeling and Substrate Docking

The homology model of *C. incerta* 15*S*‐LOX was created using AlphaFold 3.0, an advanced artificial intelligence‐based structure prediction tool, due to the low amino acid sequence identity ( < 30%) compared to available crystal structures. Molecular dynamics simulations of the docked complexes were conducted using Discovery Studio software 3.5 (San Diego, CA, USA). The substrate, 15*S*‐HETE, was docked into the active site of the model using the C‐DOCKER module. The resulting protein‐ligand complexes were further refined through energy minimization using the CHARMM force field simulator to ensure structural accuracy.

## 3 Results and Discussion

### 3.1 Discovery of a Unique Double‐Oxygenating LOX From Green Algae

A total of 468 LOX protein sequences from green algae were retrieved from the UniProt database (www.uniprot.org). Among these, 20 candidates were identified with conserved catalytic residues. From this group, 13 LOX sequences were synthesized, selecting one representative from each strain to obtain diverse green algal LOXs based on a phylogenetic tree (Supporting Information S1: Figure S1). The selected strains included *Volvox africanus* (UniProt ID: A0A8J4BD43), *Volvox carteri* f. *nagariensis* (D8U0N8), *Tribonema minus* (A0A836CEV5), *Volvox reticuliferus* (A0AJ4CY76), and *Tetradesmus obliquus* (A0A383W0J1), and eight additional LOXs.

The products were analyzed after the reaction with ARA as a substrate. Among the 13 LOXs tested, only those from *V. carteri* f. *nagariensis* (D8U0N8), *C. incerta* (A0A835WEM6), and *T. obliquus* (A0A383W0J1) produced HETE and DiHETE, identifying them as double‐oxygenating LOXs. The *C. incerta* LOX exhibited the highest DiHETE production among the green algal LOXs (Supporting Information S1: Figure S2). Therefore, this enzyme was selected from green algae LOXs as a highly active double‐oxygenating LOX.

The five metal‐binding catalytic residues and the stereoselectivity determinant residue (Coffa–Brash site) (Coffa and Brash 2004) of *C. incerta* LOX were completely conserved, aligning with those in known double‐oxygenating LOXs, including *Endozoicomonas numazuensis* 12*S*‐LOX (T. H. Kim et al. 2021), *Sphingopyxis macrogoltabida* 9*S*‐LOX (S. E. Kim et al. 2022), *A. violaceum* 15*S*‐LOX (J. Lee et al. 2020), and *Mus musculus* (mouse) 8*S*‐LOX (Jisaka et al. 2005) (Supporting Information S1: Figure S3). The amino acid sequence identities of *C. incerta* LOX with these enzymes were 23.7%, 26.4%, 24.5%, and 25.1%, respectively, indicating that *C. incerta* LOX is a LOX with a unique sequence.

### 3.2 Identification of Reaction Products Obtained From the Conversion of ARA by *C. incerta* LOX

The reaction products derived from the conversion of ARA by *C. incerta* LOX were analyzed using HPLC, LC‐MS/MS, and a polarimeter. The retention times of the ARA‐derived reaction products in the HPLC profile (Supporting Information S1: Figure S4A) were compared with those of the *A. violaceum* 15*S*‐LOX products (Supporting Information S1: Figure S4B) and the 15‐HETE standard (Supporting Information S1: Figure S4C). This analysis confirmed that the products were 15‐HETE, hepoxilin, and DiHETE, further identified as 15‐HETE, 14,15‐hepoxilin B_3_ (13‐hydroxy‐14,15‐epoxy‐eicosatrienoic acid), and 5,15‐ and 8,15‐DiHETEs using LC‐MS/MS (Supporting Information S1: Figure S5). These compounds were confirmed by referencing the Lipid Metabolites and Pathways Strategy (LIPID MAPS) database.

**Figure 2:**
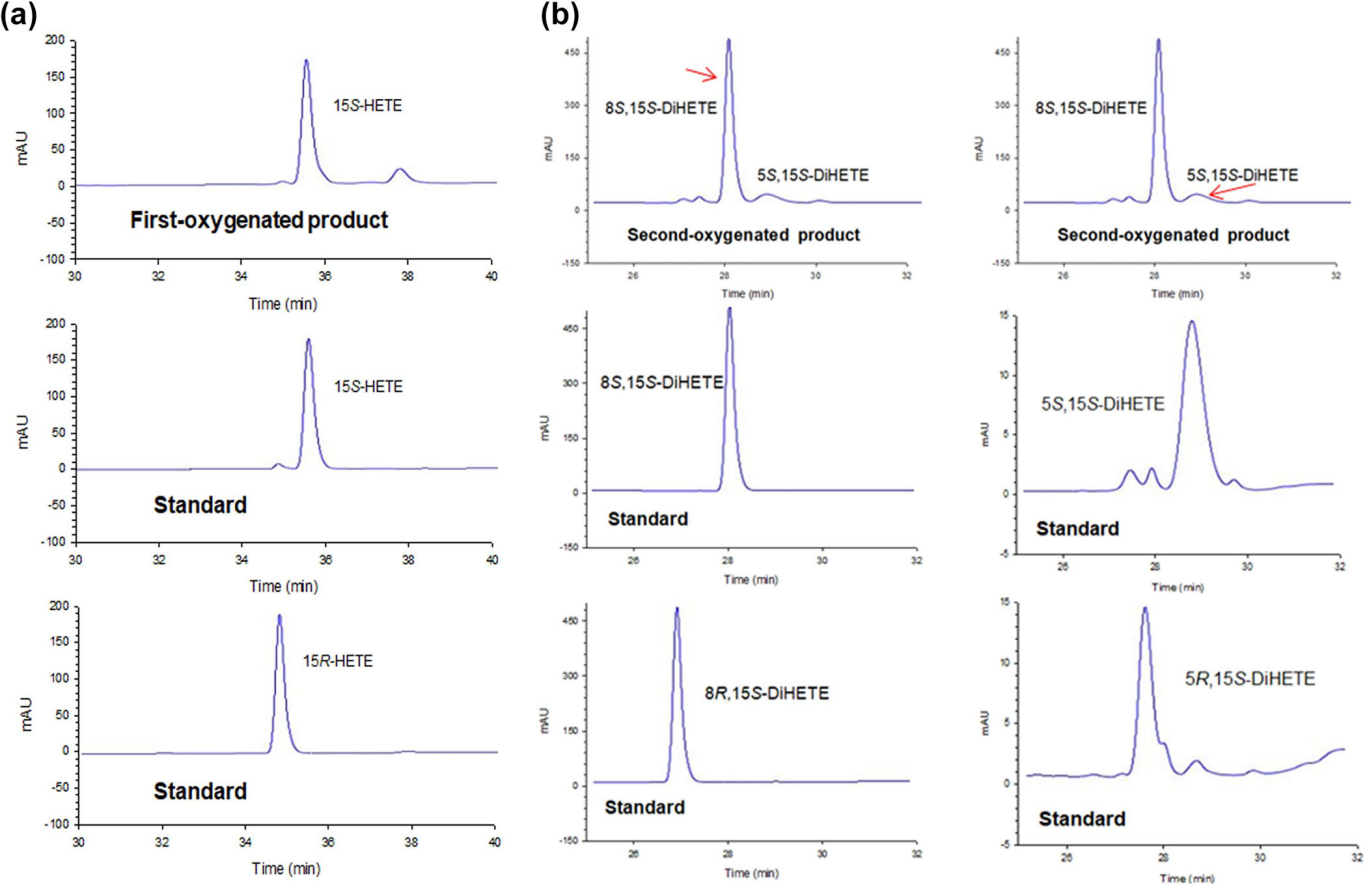
HPLC profiles with a chiral‐phase column of the reaction products obtained from converting ARA by *Chlamydomonas incerta* 15*S*‐LOX. The reaction was performed at 25°C in 50 mM HEPPS (pH 7.5) buffer containing 1.0 mM ARA and 0.5 mg/mL purified enzyme for 30 min. (a) Identification of the stereoselectivity of the first‐oxygenated product using 15*S*‐ and 15*R*‐HETE standards. The chirality of the MonoHFAs was determined at 235 nm. (b) Identification of the stereoselectivity for the second‐oxygenated products using 5*S*,15*S*‐, 5*R*,15*S*‐, 8*S*,15*S*‐, and 8*R*,15*S*‐DiHETE standards. The chirality of the DiHFAs was determined at 270 nm.

The first‐oxygenated product, 15‐HETE, produced by *C. incerta* LOX was identified as 15*S*‐HETE by comparing its retention time with those of the 15*S*‐ and 15*R*‐HETE standards using HPLC with a chiral‐phase column (Figure 2a). The retention times of the second‐oxygenated products, 5,15‐DiHETE and 8,15‐DiHETE, were identical to those of the 5*S*,15*S*‐DiHETE and 8*S*,15*S*‐DiHETE standards but differed from those of the 5*R*,15*S*‐ DiHETE and 8*R*,15*S*‐DiHETE standards. Although the optimal wavelengths for the two products are different, 5*S*,15*S*‐DiHETE and 8*S*,15*S*‐DiHETE were best separated at 270 nm, where their peaks were most clearly observed. Therefore, measurements were conducted at 270 nm (Figure 2b).

The specific rotations of the second‐oxygenated products were measured to confirm their stereoselectivity. The specific rotations for 5,15‐ and 8,15‐DiHETE products were ${\left[\alpha \right]}_{D}^{20}$ = +12.6 (0.10 g/L) and +15.8 (0.15 g/L), respectively, while the specific rotations of 5*S*,15*S*‐ and 8*S*,15*S*‐DiHETE standards were ${\left[\alpha \right]}_{D}^{20}$ = +12.6 (0.12 g/L) and +15.7 (0.14 g/L), respectively. These results confirm that the products are 5*S*,15*S*‐ and 8*S*,15*S*‐DiHETEs. Additionally, the specific rotations of 5*R*,15*R*‐DiHETE and 8*R*,15*R*‐DiHETE standards were ${\left[\alpha \right]}_{D}^{20}$ = −14.7 (0.13 g/L) and −18.2 (0.19 g/L), respectively. These findings further validate the stereo‐configuration of the products. Therefore, *C. incerta* LOX was characterized as a double‐oxygenating 15*S*‐LOX capable of simultaneously producing 5*S*,15*S*‐DiHETE and 8*S*,15*S*‐DiHETE from ARA.

### 3.3 Biochemical Characterization of the Purified 15*S*‐LOX From *C. incerta*

The molecular mass of the purified 15*S*‐LOX from *C. incerta* was determined by SDS‐PAGE analysis as approximately 110 kDa (Supporting Information S1: Figure S6A), which closely corresponded to the calculated molecular mass of 109,248 kDa based on its 1016 amino acid residues and additional six histidine residues. Gel‐filtration chromatography indicated that the native protein formed a dimer with an estimated total molecular mass of 219 kDa (Supporting Information S1: Figure S6B).

The activity of 15*S*‐LOX from *C. incerta*, which converts ARA into DiHETEs (including 5*S*,15*S*‐DiHETE and 8*S*,15*S*‐DiHETE) were evaluated under varying pH and temperature conditions. Maximal activity occurred at pH 8.0 and 35°C, while the highest proportion of 8*S*,15*S*‐DiHETE was observed at pH 7.5 and 35°C (Supporting Information S1: Figure S7).

Substrate specificity was tested using ARA, EPA, and DHA (Table 1). The specific activities and catalytic efficiency (*k*_cat_ */K*_m_) followed the order ARA > DHA > EPA, confirming *C. incerta* 15*S*‐LOX as an ARA‐selective enzyme. Comparisons of kinetic parameters for DHA with those of other double‐oxygenating LOXs revealed the following order of catalytic efficiency (*k*_cat_/*K*_m_): *S. cellulosum* 15*R*‐LOX>*C. incerta* 15*S*‐LOX>*A. violaceum* 15*S*‐LOX>*E. numazuensis* 12*S*‐LOX>*S. macrogoltabida* 9*S*‐LOX.

**Table 1:** Specific activities and kinetic parameters of the double‐oxygenating LOXs.

Organism	Type	Substrate	Product	Specific activity (μmol/min/mg)	*K*_m_ (μM)	*k*_cat_ (1/s)	*k*_cat_ */K*_m_ (1/s/μM)	References
*C. incerta*	15*S*‐LOX	ARA	15*S*‐HETE	22.7 ± 2.5	101 ± 0.02	149 ± 0.2	1.47 ± 0.1	This study
		EPA	15*S*‐HEPA	17.7 ± 2.9	191 ± 0.1	143 ± 0.2	0.75 ± 0.1	
		DHA	17*S*‐HDHA	18.5 ± 2.9	98.5 ± 0.1	121 ± 0.2	0.98 ± 0.1	
*S. macrogoltabida*	9*S*‐LOX	ARA	9*S*‐HETE	1.58 ± 0.02	23.4 ± 1.1	12.1 ± 0.2	0.52 ± 0.02	S. E. Kim et al. (2022)
		EPA	9*S*‐HEPA	1.09 ± 0.03	9.12 ± 0.4	3.35 ± 0.3	0.37 ± 0.005	
		DHA	11*S*‐HDHA	1.36 ± 0.08	9.85 ± 0.5	3.75 ± 0.5	0.38 ± 0.03	
*E. numazuensis*	12*S*‐LOX	ARA	12*S*‐HETE	24.7 ± 0.2	44.2 ± 0.2	107 ± 1.4	2.49 ± 0.3	T. H. Kim et al. (2021)
		EPA	12*S*‐HEPA	15.3 ± 0.2	35.2 ± 0.2	33.2 ± 0.8	0.94 ± 0.2	
		DHA	14*S*‐HDHA	12.9 ± 0.1	47.9 ± 0.1	27.3 ± 0.6	0.57 ± 0.2	
*S. cellulosum*	15*R*‐LOX	ARA	15*R*‐HETE	64.5 ± 0.2	84.5	337	3.99	T. E. Lee, Ko, Shin, et al. (2024)
		EPA	15*R*‐HEPA	36.0 ± 0.3	113.3	163	1.43	
		DHA	17*R*‐HDHA	55.8 ± 0.4	70.3	133	1.89	
*A. violaceum*	15*S*‐LOX	ARA	15*S*‐HETE	28.4 ± 0.2	68.5	164	2.39	J. Lee et al. (2020)
		EPA	15*S*‐HEPA	15.4 ± 0.3	58.2	32.6	0.56	
		DHA	17*S*‐HDHA	18.8 ± 0.2	33.7	31.8	0.94	

*Note:* Specific activities and kinetic parameters were determined by measuring the increase in absorbance at 234 nm as hydroxy fatty acid amounts by using a spectrometer for the reaction solutions derived from polyunsaturated fatty acids by double‐oxygenating LOXs.

Abbreviations: ARA, arachidonic acid; DHA, docosahexaenoic acid; EPA, eicosapentaenoic acid; HDHA, hydroxy docosahexaenoic acid; HEPA, hydroxy eicosapentaenoic acid; HETE, hydroxy eicosatetraenoic acid; LOX, lipoxygenase.

### 3.4 Optimization of the Reaction Conditions for Producing the DiHDHAs, RvD5, and PDX, Using *E. coli* Expressing *C. incerta* 15*S*‐LOX

**Figure 3:**
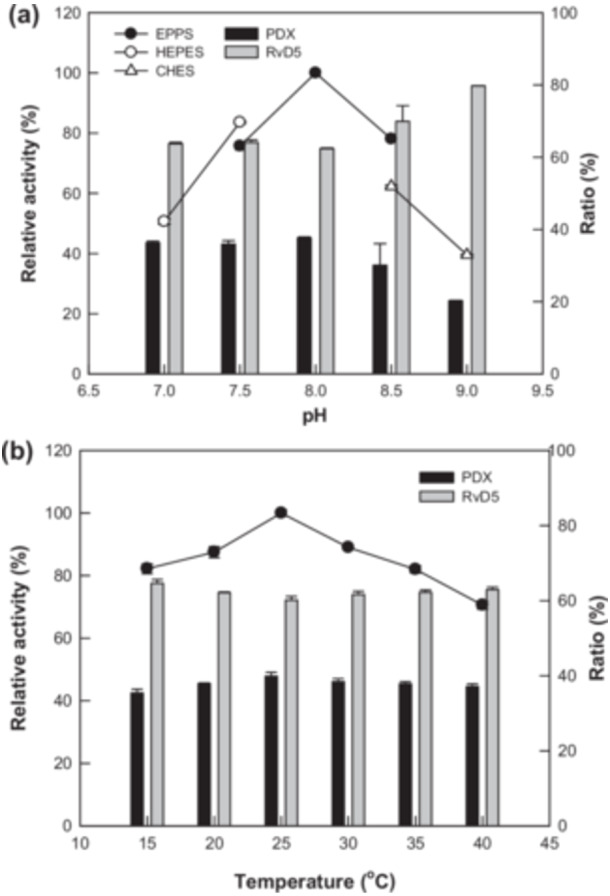
Effects of pH and temperature on 5*S*,15*S*‐DiHDHA (RvD5) and 8*S*,15*S*‐DiHDHA (PDX) production from DHA by *Escherichia coli* expressing *Chlamydomonas incerta* 15*S*‐LOX. (a) Effect of pH on the production of RvD5 and PDX. (b) Effect of temperature on the production of RvD5 and PDX.

**Figure 4:**
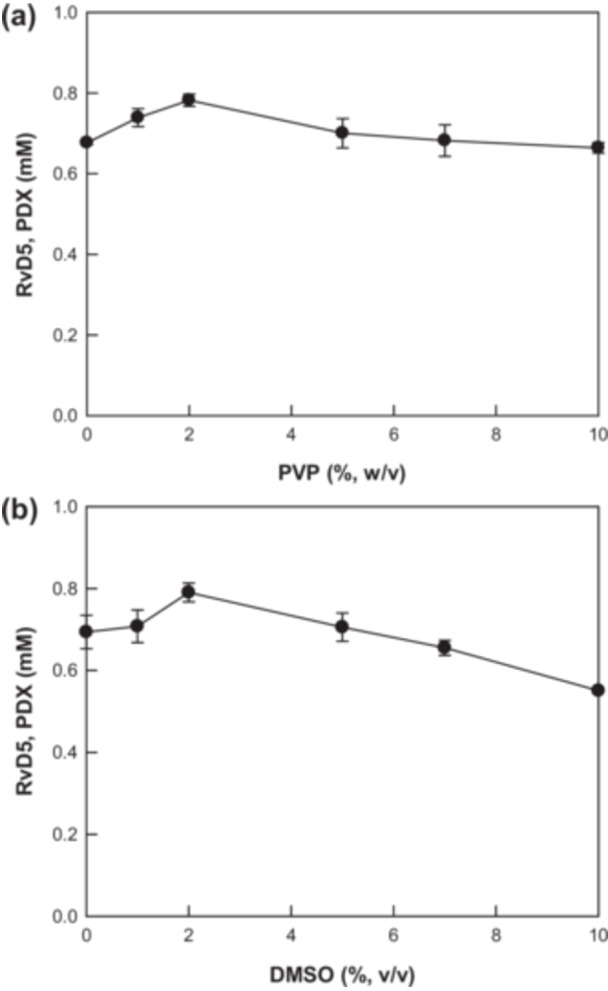
Optimization of PVP and DMSO concentrations for DiHDHA production from DHA by *Escherichia coli* cells expressing *Chlamydomonas incerta* 15*S*‐LOX. (a) Optimization of PVP for DiHDHA production. (b) Optimization of DMSO for DiHDHA production.

**Figure 5:**
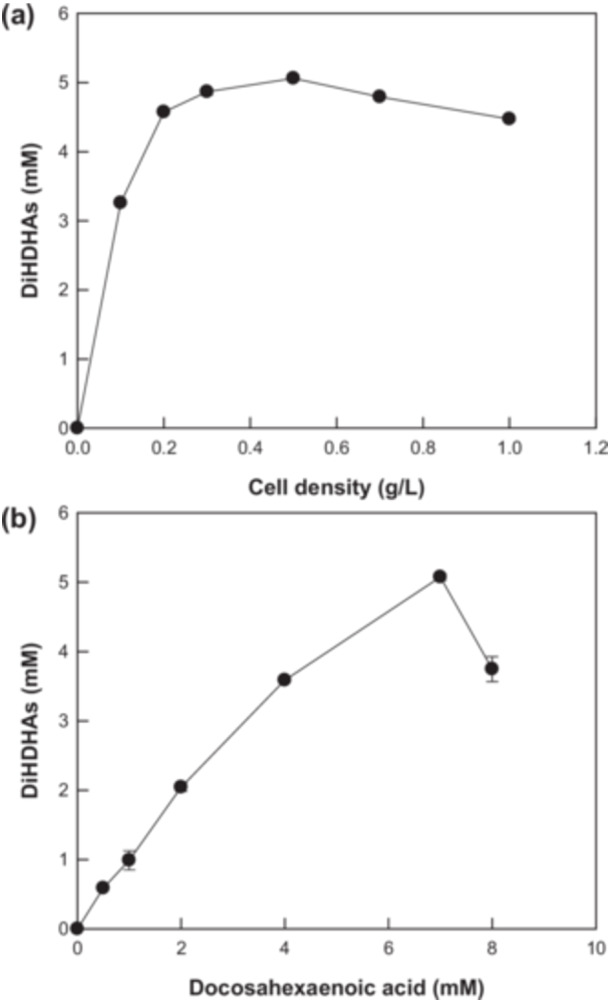
Optimization of cell and DHA concentrations for DiHDHA production from DHA by *Escherichia coli* cells expressing *Chlamydomonas incerta* 15*S*‐LOX. (a) Optimization of cell concentration for DiHDHA production. (b) Optimization of DHA concentration for DiHDHA production.

The conversion of DHA into DiHDHAs, specifically RvD5 and PDX, was performed using *E. coli* expressing *C. incerta* 15*S*‐LOX by varying the pH and temperature (Figure 3). The maximal DiHDHA production and highest PDX proportion was achieved at pH 8.0 and 25°C. PVP and DMSO can enhance DiHDHA production by improving the substrate solubility (J. Lee et al. 2023). Their concentrations were optimized and determined as 2.0% (w/v) PVP and (v/v) 2.0% DMSO (Figure 4). The cell density and DHA concentration were also optimized, and the maximal production of DiHDHAs was achieved at 0.5 g/L cells (Figures 4a) and 7.0 mM DHA (Figure 5b).

### 3.5 Efficient One‐Step Production of DiHDHAs, RvD5, and PDX, Using *E. coli* Expressing *C. incerta* Double‐Oxygenating 15*S*‐LOX

**Figure 6:**
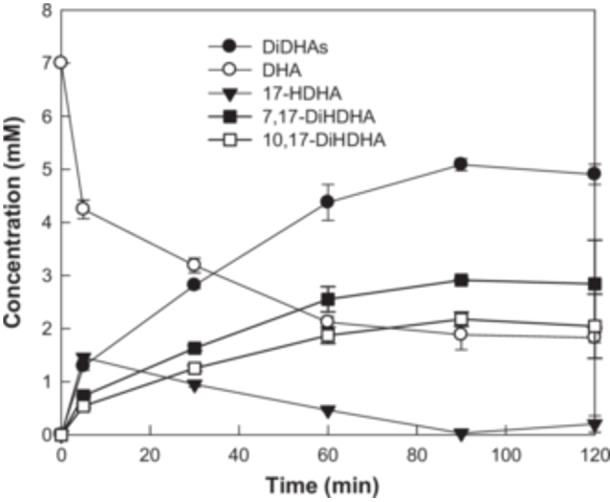
Biotransformation of DHA into RvD5 and PDX via 17‐HDHA using *Escherichia coli* cells expressing *Chlamydomonas incerta* 15*S*‐LOX.

Under the optimized conditions, the cells produced 5.09 mM (1.83 g/L) of DiHDHAs, comprising 2.91 mM (1.05 g/L) RvD5 and 2.18 mM (0.78 g/L) PDX from 7.0 mM DHA in 90 min, with a total 5.09 mM (1.83 g/L) and a total conversion yield of 79.6% (w/w) (Figure 6). The specific productivity was calculated as 6.79 mmol/h/g, and the volumetric productivity was 3.39 mM/h.

The production of regio‐ and stereo‐selective DiHDHAs from DHA using LOXs expressed in *E. coli* is summarized in Table 2. DiHDHA concentrations and productivities from the one‐step reaction with double‐oxygenating LOXs were significantly higher than those achieved via a two‐step process involving two distinct regioselective single‐oxygenating LOXs. Among the tested LOXs, *S. macrogoltabida* 9*S*‐LOX expressed in *E. coli* displayed the highest DiHDHA production, followed by *C. incerta* 15*S*‐LOX, *S. cellulosum* 15*R*‐LOX, *A. violaceum* 15*S*‐LOX, and *E. numazuensis* 12*S*‐LOX.

**Table 2:** Production of regio‐ and stereo‐selective DiHDHAs from DHA by LOXs expressed in *E. coli* cells.

Reaction	LOX expressed in *E. coli*	Substrate (mM)	Product (mM)	Specific productivity (mmol/h/g)	Volumetric productivity (mM/h)	Molar conversion (%)	References
One step	*C. incerta* 15*S*‐LOX	DHA (7.0)	RvD5, PDX (5.1)	6.78 ± 0.21	3.39 ± 0.1	74.1 ± 0.15	This study
			RvD5 (2.9)	3.88 ± 0.07	1.94 ± 0.04	41.6 ± 0.06	
			PDX (2.2)	2.91 ± 0.13	1.45 ± 0.06	31.1 ± 0.09	
	*A. violaceum* 15*S*‐LOX	DHA (3.0)	RvD5 (2.8)	2.80	2.80	93.3	J. Lee et al. (2020)
	*S. macrogoltabia* 9*S*‐LOX	DHA (6.0)	11*S*,17*S*‐DiHDHA (5.2)	0.75 ± 0.004	5.24 ± 0.026	87.3 ± 0.1	Oh et al. (2022)
	*E. numazuensis* 12*S*‐LOX	DHA (3.0)	7*S*,14*S*‐DiHDHA (1.5)	0.58 ± 0.001	1.16 ± 0.003	51.5 ± 0.7	T. H. Kim et al. (2021)
	*S. cellulosum* 15*R*‐LOX	DHA (6.0)	7*R*,17*R*‐DiHDHA (4.8)	2.11 ± 0.006	3.17 ± 0.009	79.1 ± 0.01	T. E. Lee, Ko, Shin, et al. (2024)
Two steps	Mouse 8*S*‐LOX	DHA (1.0)	10*S*‐HDHA (0.43)	0.02 ± 0.001	0.07 ± 0.002	43.0 ± 1.0	Shin et al. (2022)
	*B. thailandensis* 15*S*‐LOX	10*S*‐HDHA (0.43)	PDX (0.30)	0.013 ± 0.001	0.06 ± 1.0	69.8 ± 2.3	
	*P. homomalla* 8*R*‐LOX	DHA (2.0)	10*R*‐HDHA (1.4)	0.53 ± 0.01	2.10 ± 0.07	70.0 ± 2.3	J. Lee, Ko, Park, et al. (2024)
	*A. violaceum* 15*S*‐LOX	10*R*‐HDHA (1.4)	10*R*,17*S*‐DiHDHA (1.2)	1.80 ± 0.03	2.40 ± 0.03	85.7 ± 1.3	

*Note:* Products were detected using high‐performance liquid chromatography by measuring the increase in absorbance at 270 nm as dihydroxy fatty acid amounts for the reaction solutions derived from using substrates.

Abbreviations: DHA, docosahexaenoic acid; DiHDHA, dihydroxy docosahexaenoic acid; HDHA, hydroxy docosahexaenoic acid; LOX, lipoxygenase; PDX, protectin DX; RvD5, resolvin D5.

The concentration and specific productivity of RvD5 produced by *E. coli* expressing *C. incerta* 15*S*‐LOX produced RvD5 were 1.04‐ and 1.39‐fold higher, respectively, than those produced by *E. coli* expressing *A. violaceum* 15*S*‐LOX (J. Lee et al. 2023). The PDX concentration from the one‐step biocatalytic process was 7.3‐fold higher than that achieved through two‐step processes using *M. musculus* (mouse) 8*S*‐LOX and *Burkholderia thailandensis* 15*S*‐LOX (Shin et al. 2022). Additionally, PDX specific and volumetric productivities were 223‐ and 24.2‐fold higher, respectively, compared to the two‐step process. Although the concentrations of PDX were obtained at the optimal concentrations for each reaction, these comparisons do not account for differences in substrate concentrations between the one‐step and two‐step processes. These findings exhibited the efficiency of *E. coli* expressing *C. incerta* 15*S*‐LOX as a biocatalyst for PDX production. This study represents the first quantitative production of PDX via a one‐step biocatalytic process using *C. incerta* 15*S*‐LOX and demonstrates its strong potential for RvD5 production.

### 3.6 Identification of Residues Modulating Regioselectivity in the Second Oxygenation Step of *C. incerta* Double‐Oxygenating 15*S*‐LOX

**Figure 7:**
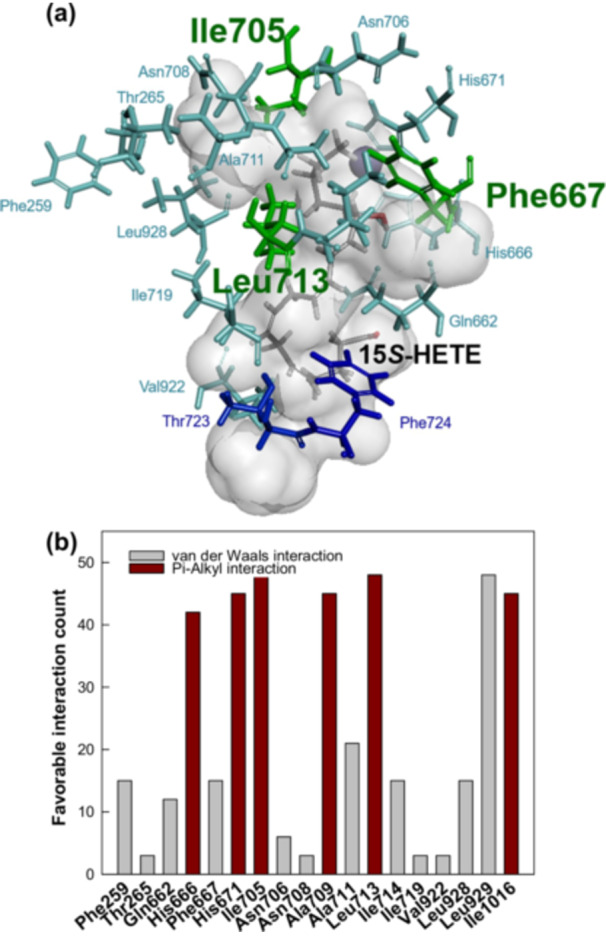
Interaction analysis of the active‐site residues in *Chlamydomonas incerta* 15*S*‐LOX with the substrate 15*S*‐HETE using the docking model. (a) Docking model for the interactions between 15*S*‐HETE and the active‐site residues. Regioselective residues for the first and second oxygenation steps, substrate‐interacting residues, and the ligand are indicated in the dark blue (Thr723 and Phe724), green (Phe667, Ile705, and Leu713), light blue, and dark gray, respectively. (b) Interaction counts of favorable residues in the active site to 15*S*‐HETE in 50 docking models.

The Leu429 and Leu430 residues of *A. violaceum* 15*S*‐LOX modulate the regioselectivity of the first oxygenation step (J. Lee et al. 2020; J. Lee, Ko, Park, et al. 2024). These residues corresponded to Thr723 and Phe724 in *C. incerta* 15*S*‐LOX, suggesting their critical role in modulating regioselectivity for the first oxygenation step (Figure 7a).

Docking analysis of *C. incerta* 15S‐LOX identified residues with more than 40 favorable interactions with the ligand 15*S*‐HETE in 50 docking models. These residues included His666, Phe667, His671, Ile705, Ala709, Leu713, Leu929, and Ile1016 (Figure 7b). Among these, the catalytic His666, His671, Ile1016, and the Coffa–Brash site Ala709 were excluded, while the Ala711 and Leu929 variants exhibited no second oxygenation activity. Consequently, Phe667, Ile705, and Leu713 were selected for further investigation.

These residues were substituted with small (Ala and Gly) and large (Phe and Trp) amino acids to assess their roles in modulating regioselectivity. The ratios of 5*S*,15*S*‐DiHETE and 8*S*,15*S*‐DiHETE produced by the variants were analyzed (Table 3). The wild‐type enzyme exhibited a product ratio of 57.0% 5*S*,15*S*‐DiHETE and 43.0% 8*S*,15*S*‐DiHETE, while the F667A variant exhibited a decreased proportion of 8*S*,15*S*‐DiHETE (9.0%) and F667W caused no significant changes, likely due to the similar size of Phe and Trp. The I705G, I705A, L713G, and L713A variants increased the proportion of 8*S*,15*S*‐DiHETE, whereas I705F and L713F exhibited slightly decreased proportions. Notably, the L713A variant produced the highest proportion of 8*S*,15*S*‐DiHETE (74.6%) but reduced the specific activity for converting ARA into DiHETEs to 18.9% of the wild‐type enzyme. This indicates that while regioselectivity was improved, the overall catalytic activity of the enzyme was reduced.

**Table 3:** Specific activities of the wild‐type and variant 15*S*‐LOXs from *C. incerta* in converting ARA to DiHETEs and the regio‐isomeric ratios of the resulting DiHETEs.

Enzyme	Specific activity (μmol/min/mg)	5*S*,15*S*‐DiHETE (%)	8*S*,15*S*‐DiHETE (%)
Wild‐type	61.3 ± 1.98	57.0	43.0
F667A	6.87 ± 0.07	91.0	9.0
F667W	3.13 ± 0.07	58.5	41.5
I705G	3.47 ± 0.20	42.8	57.2
I705A	6.30 ± 0.19	35.2	64.8
I705F	2.07 ± 0.06	61.0	39.0
L713G	14.8 ± 0.80	25.4	74.6
L713A	11.6 ± 0.66	25.3	74.7
L713F	2.07 ± 0.20	61.2	38.7

*Note:* Products were detected using high‐performance liquid chromatography by measuring the increase in absorbance at 270 nm as dihydroxy fatty acid amounts for the reaction solutions derived from HFA as a substrate.

Abbreviation: DiHETE, dihydroxy eicosatetraenoic acid.

## 4 Conclusions

In summary, a unique double‐oxygenating 15*S*‐LOX from *C. incerta* was identified and characterized for the efficient production of two SPMs, RvD5 and PDX. Under optimized conditions, including pH, temperature, and PVP, DMSO, cell, and substrate concentrations, were optimized using *E. coli* expressing *C. incerta* 15*S*‐LOX. Under optimized reaction conditions, the one‐step biocatalytic process produced high RvD5 (2.91 mM) and PDX (2.18 mM) concentrations in 90 min. This one‐step biocatalytic process offers significant advantages for PDX production regarding simplicity, productivity, and product concentration compared to previously reported two‐step biocatalytic processes using two distinct single‐oxygenating LOXs. Structural analysis identified the key residues Phe667, Ile705, and Leu713 as regioselectivity modulators in the second oxygenation step. This study provides the first quantitative report of the simultaneous production of RvD5 and PDX, demonstrating the efficiency and potential of double‐oxygenating LOX as an industrially viable biocatalyst.

## Author Contributions

Hyun‐Ah Park and Deok‐Kun Oh conceived the study and designed the experiments. Hyun‐Ah Park mainly performed experiments, including product identification, bioconversion condition optimization, MonoHFA bioconversion, and HPLC analysis. Jin Lee performed. Hyun‐Ah Park, Jin Lee, and Deok‐Kun Oh reviewed the data and prepared the manuscript. All authors have read and approved the manuscript.

## Conflicts of Interest

The authors declare no conflicts of interest.

## Supporting information

CI_LOX_BB_SI_ver2.

## Data Availability

The data that support the findings of this study are available from the corresponding author upon reasonable request.
