# Supplementary material for: Efficient One‐Step Production of 7S,17S‐ and 10S,17S‐Dihydroxydocosahexaenoic Acids by a Double‐Oxygenating 15S‐Lipoxygenase From Chlamydomonas incerta
Source: Biotechnol Bioeng. 2025 Apr 15;122(7):1747–58. doi: 10.1002/bit.28997 (PMC12152530; doi:10.1002/bit.28997)
Supplement: Supplementary file 1 — CI_LOX_BB_SI_ver2. [file BIT-122-1747-s001.docx]

**Supporting Information**

**Supplement Figures**

**
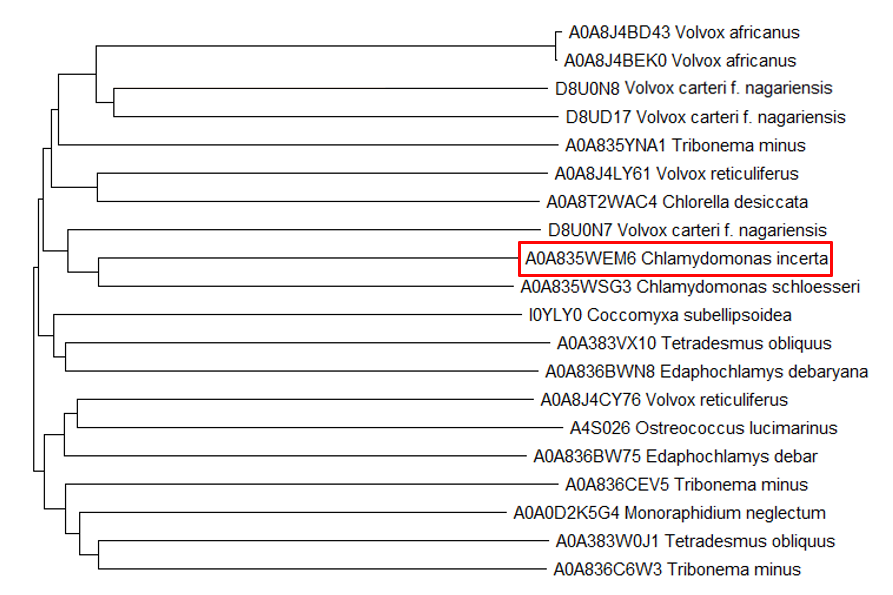
**

**Figure S1.** Phylogenetic tree of green algal LOXs in this study based on amino acid sequences. The evolutionary analysis was conducted using MEGA11. One representative from each strain was included to obtain diversity in LOX selection, while seven additional putative LOXs from the same strains were excluded. These excluded LOXs included those from *V. africanus* (UniProt ID: A0A8J4BEK0), *V. carteri* f. *nagariensis* (D8UD17 and D8U0N7), *T. minus* (A0A836BW75 and A0A836C6W3), *V. reticuliferus* (A0AJ4CY61), and *T. obliquus* (A0A383VX10).


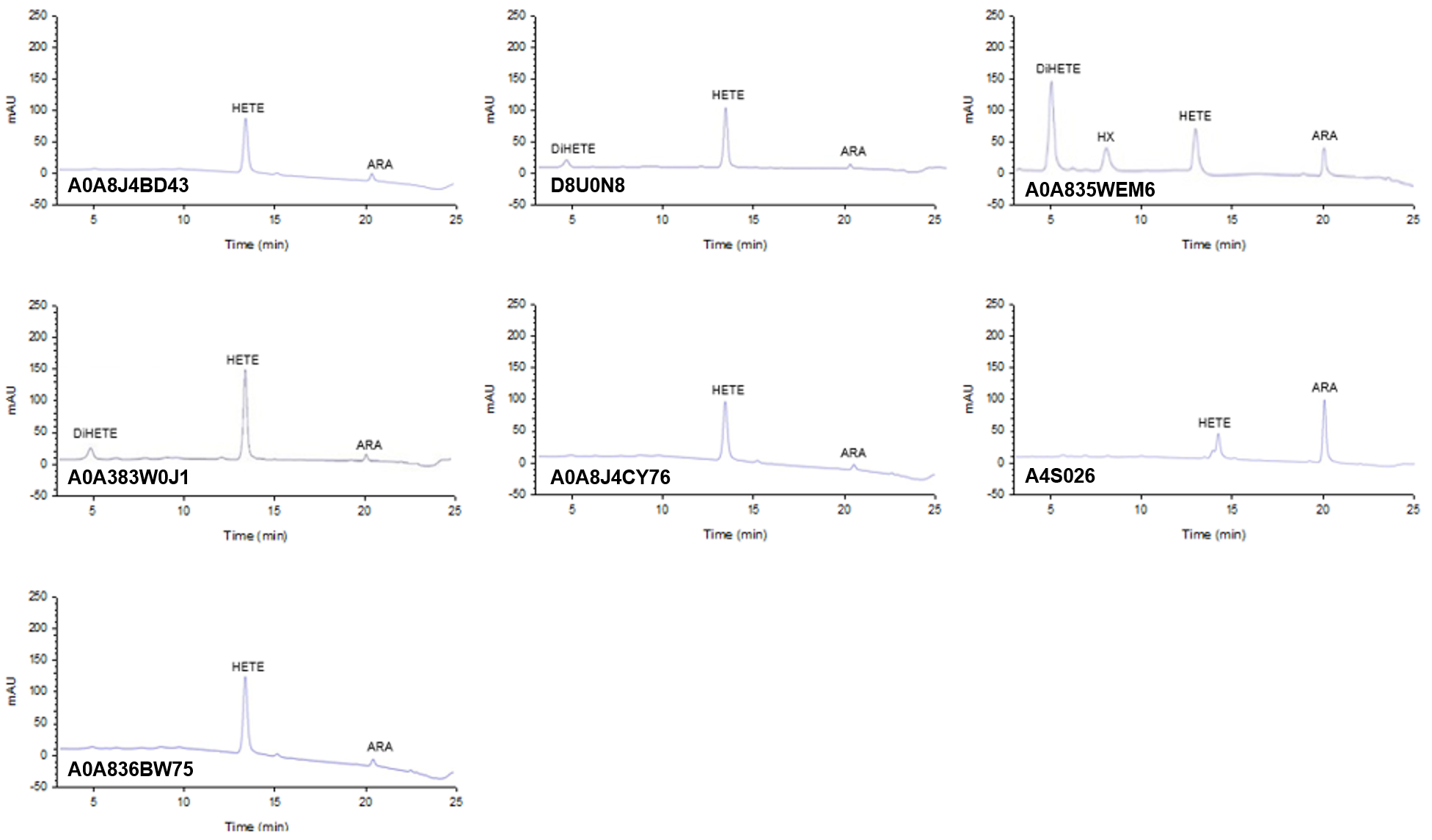


**Figure S2.** HPLC profiles at 202 nm of products obtained from the conversion of ARA by the seven LOXs. LOXs from *V. carteri* f. *nagariensis* (D8U0N8), *C. incerta* (A0A835WEM6), and *T. obliquus* (A0A383W0J1) produced HETE and DiHETE, while LOXs from *V. africanus* (A0A8J4BD43), *V. reticuliferus* (A0AJ4CY76), *O. lucimarinus* (A4S026), and *E. debaryana* (A0A836BW75) produced only HETE. No detectable products were observed for the remaining six LOXs. The reactions were performed at 25 °C in 50 mM HEPPS (pH 7.5) containing 1.0 mM ARA, 0.5 mg/mL purified enzyme, and 200 mM cysteine as the reducing agent for 30 min.


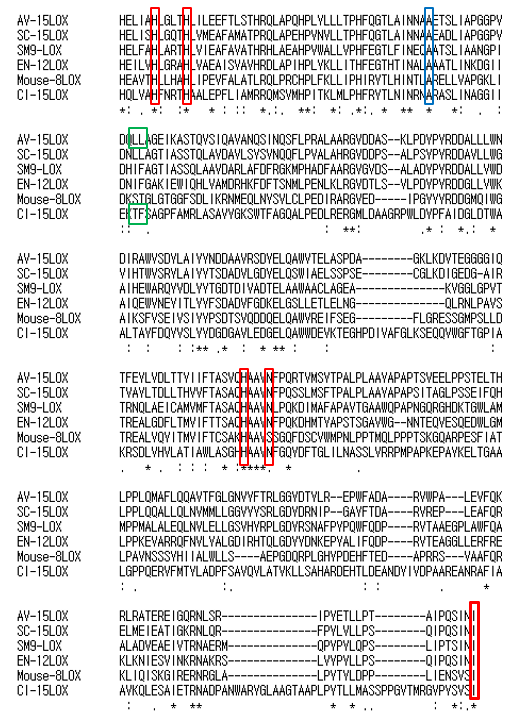


**Figure S3.** Amino acid alignment of *C. incerta* 15*S*-LOX with other double-oxygenating LOXs, including *S. macrogoltabida* 9*S*-LOX, *E. numazuensis* 12*S*-LOX, *A. violaceum* 15*S*-LOX, *S. cellulosum* 15*R*-LOX, and *M. musculus* 8*S*-LOX. The alignment revealed completely conserved catalytic residues (His666, His671, His860, Asn864, and Ile1016). The catalytic residues, the Coffa-Brash site as a stereoselective determinant (Ala 709), and the regioselective determinants for the first oxygenation step (Thr723 and Phe724) are highlighted in red, blue, and green, respectively.

**(a)**

**
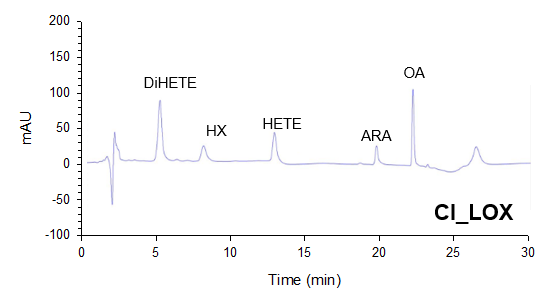
**

**(b)**

**
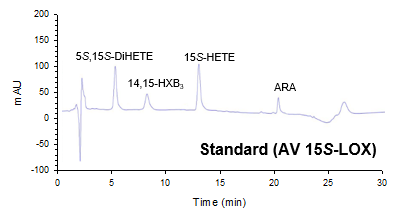
**

**(c)**


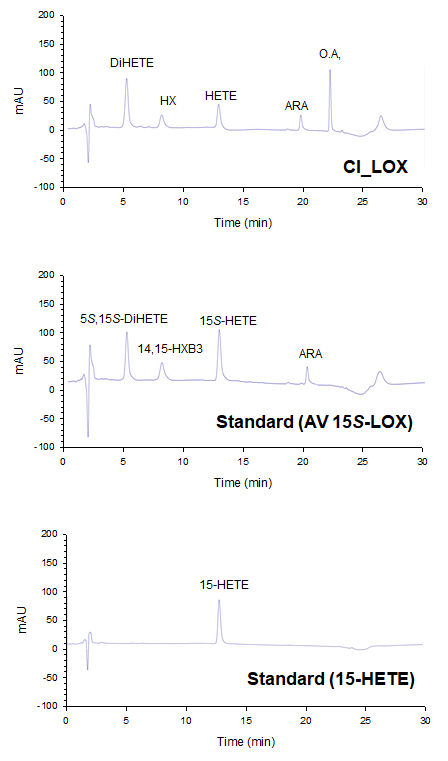


**Figure S4**. HPLC profiles of the reaction products obtained from the conversion of ARA by 15*S*-LOX from *C. incerta* with those produced by *A. violaceum* 15*S*-LOX and the 15-HETE standard. (a) HPLC profile of products obtained from the conversion of ARA by 15*S*-LOX from *C. incerta*. The reaction was performed at 25°C in 50 mM HEPPS (pH 7.5) containing 1.0 mM ARA and 0.5 mg/mL purified enzyme for 30 min. (b) HPLC profile of products obtained from the conversion of ARA by 15*S*-LOX from *A. violaceum*. The reaction was performed at 20°C in 50 mM HEPPS (pH 8.5) containing 1.0 mM ARA and 0.05 mg/mL purified enzyme for 30 min. (c) HPLC profile of the 15-HETE standard. HX, hepoxilin; OA, oleic acid; 14,15-HXB_3_ (14,15-hepoxilin B_3_), 13-hydroxy-14,15-epoxyeicosatetraenoic acid.

**(a)**

**
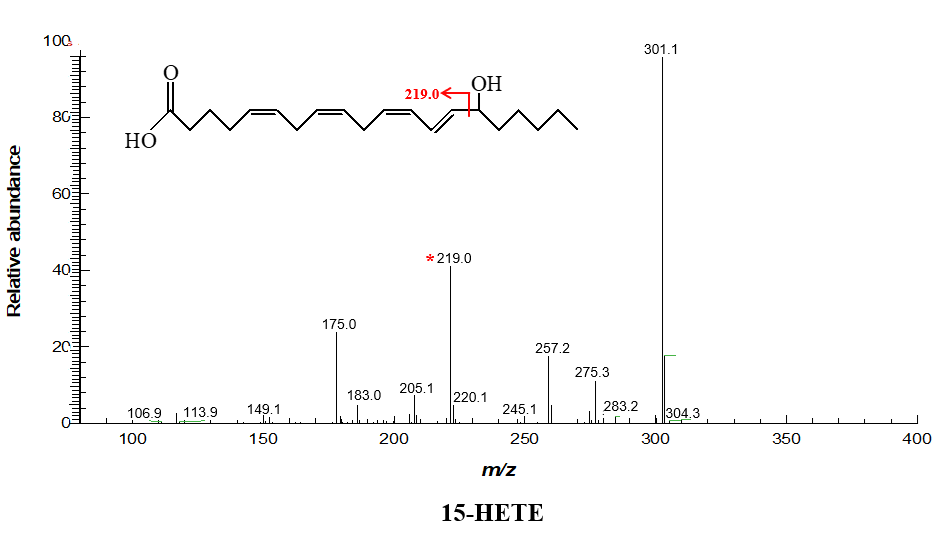
**

**(b)**

**
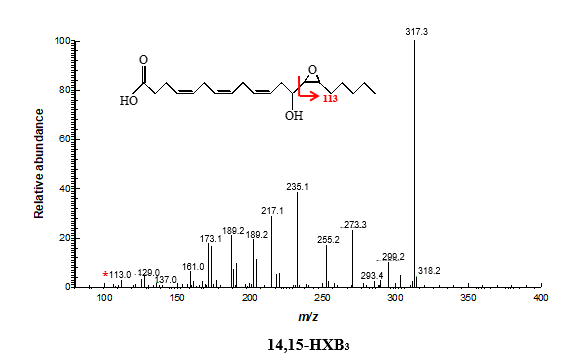
**

**(c)**

**
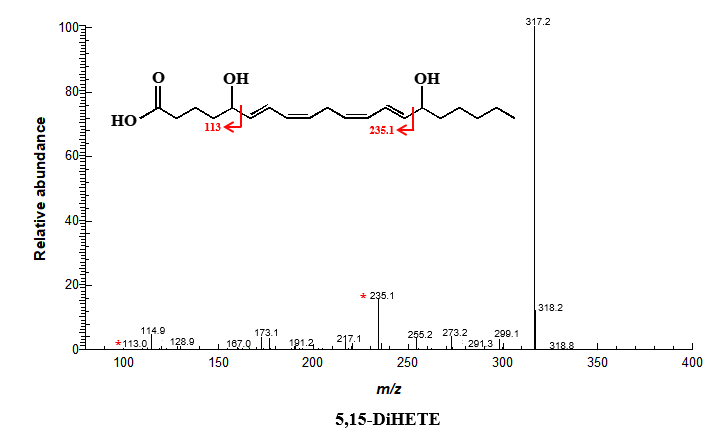
**

**(d)**


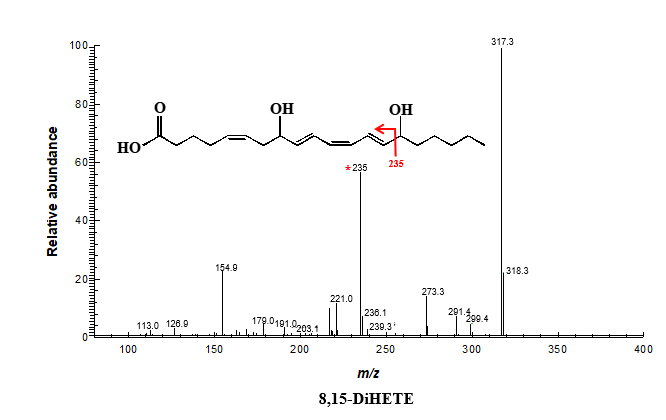


**Figure S5**. LC-MS/MS chromatograms of the reaction products obtained from the conversion of ARA by 15*S*-LOX from *C. incerta*. Red arrows indicate key fragments associated with the hydroxyl group. (a) 15-HETE. (b) 14,15-HXB3. (c) 5,15-DiHETE. (d) 8,15-DiHETE.

**(a)**

**
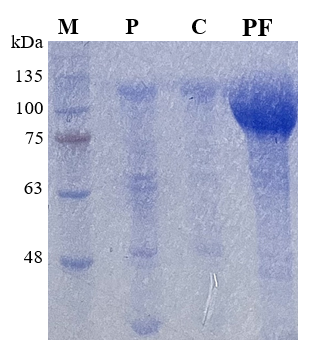
**

**(b)**

**
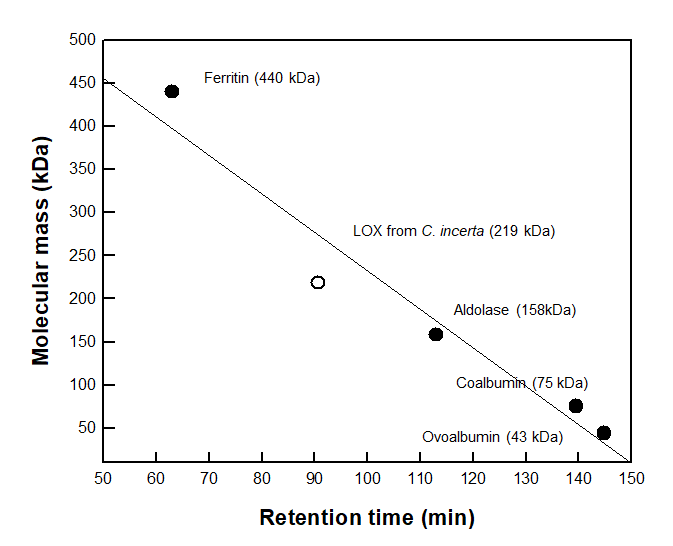
**

**Figure S6**. Determination of the molecular mass of purified *C. incerta* 15*S*-LOX using SDS-PAGE and gel-filtration chromatography. (a) SDS-PAGE analysis of *C. incerta* 15*S*-LOX. M, protein marker; P, pellet; C, crude extract; PF, purified *C. incerta* 15*S*-LOX. (b) Total molecular mass of purified *C. incerta* 15*S*-LOX using gel-filtration chromatography. The reference proteins used were ovalbumin (44 kDa), conalbumin (75 kDa), aldolase (158 kDa), and ferritin (440 kDa). The purified *C. incerta* 15*S*-LOX was eluted at a retention time corresponding to 219 kDa.

**(a)**

**(b)**

**Figure S7**. Effects of pH and temperature on the production of 5*S*,15*S*-DiHETE and 8*S*,15*S*-DiHETE from ARA by *C. incerta* 15*S*-LOX. (a) Effect of pH on 5*S*,15*S*-DiHETE and 8*S*,15*S*-DiHETE production. The reactions were performed at 35 °C in 50 mM HEPES (pH 7.0−7.5), 50 mM HEPPS (pH 7.5−8.5), and 50 mM CHES (pH 8.5−9.0) buffers containing 1.0 mM ARA, 0.5 mg/mL enzyme, and 200 mM cysteine as a reducing agent for 30 min by varying the pH from 7.0 to 9.0. (b) Effect of temperature on 5*S*,15*S*-DiHETE and 8*S*,15*S*-DiHETE production. The reactions were performed in 50 mM HEPPS (pH 7.5) buffer containing 1.0 mM ARA, 0.5 mg/mL enzyme, and 200 mM cysteine as a reducing agent for 30 min by varying the temperature from 20 °C to 40 °C.
